# Effectiveness of Virtual Yoga for Chronic Low Back Pain: A Randomized Clinical Trial

**DOI:** 10.1001/jamanetworkopen.2024.42339

**Published:** 2024-11-01

**Authors:** Hallie Tankha, Devyn Gaskins, Amanda Shallcross, Michael Rothberg, Bo Hu, Ning Guo, Eric J. Roseen, Stephen Dombrowski, Judi Bar, Renee Warren, Holly Wilgus, Piper Tate, Johanna Goldfarb, Victoria Garcia Drago, Robert Saper

**Affiliations:** 1 Department of Wellness and Preventive Medicine, Primary Care Institute, Cleveland Clinic, Cleveland, Ohio; 2 Department of Internal Medicine, Primary Care Institute, Cleveland Clinic, Cleveland Ohio; 3 Quantitative Health Sciences, Lerner Research Institute, Cleveland Clinic, Cleveland, Ohio; 4 Section of General Internal Medicine, Department of Medicine, Boston University Chobanian & Avedisian School of Medicine and Boston Medical Center, Boston, Massachusetts; 5 Cleveland Clinic Innovations, Cleveland Clinic, Cleveland, Ohio; 6 Victoria Garcia Drago Yoga and Wellness, Oakland, California

## Abstract

**Importance:**

Chronic low back pain (CLBP) is a common condition with substantial impact on patients, in the form of physical and emotional suffering; health care costs; productivity; and health care professional burden. Although clinical guidelines recommend use of nonpharmacologic treatments first, such as yoga, there is a gap between guidelines and implementation in clinical practice.

**Objective:**

To compare the effects of virtual yoga classes vs a wait-list control on CLBP intensity, back-related function, sleep quality, and pain medication use.

**Design, Setting, and Participants:**

Single-blinded, 24-week, 2-arm, randomized clinical trial conducted from May 3, 2022, through May 23, 2023, comparing live streamed yoga classes (the yoga now group) with a wait-list control (the yoga later group, in which participants were offered the virtual intervention after the study but without assessments) among adults with CLBP. Adults 18 to 64 years of age with CLBP were recruited from the Cleveland Clinic Employee Health Plan, a large health system self-insured health plan. Inclusion criteria included a mean LBP intensity score of at least 4 on an 11-point numerical rating scale (scores ranged from 0 to 10, with higher scores indicating worse pain) and daily back pain interference about half or more of the days.

**Intervention:**

Twelve consecutive weekly, 60-minute, virtual, live streamed hatha yoga group classes.

**Main Outcomes and Measures:**

Coprimary outcomes were mean pain intensity in the previous week on the 11-point numerical rating scale and back-related function as assessed using the 23-point modified Roland Morris Disability Questionnaire ([RMDQ], with higher scores reflecting poorer function) at 12 weeks. Secondary and exploratory outcomes included these measures at 24 weeks, pain medication use, and PROMIS sleep quality assessed using the Sleep Disturbance Short Form 8a, item 1 (scores ranged from 0 to 4, with higher scores reflecting better sleep quality). Analyses followed the intention-to-treat principle.

**Results:**

Among 140 participants enrolled (yoga now = 71; yoga later = 69), the mean (SD) age was 47.8 (11.7) years and most were female (113 [80.7%]) and college-educated (103 [73.5%]). Mean (SD) baseline pain intensity (5.7 [1.5]) and RMDQ ([12.1 [4.4]) scores reflected moderate back pain and impairment. At week 12, yoga now compared with yoga later had greater reductions in mean pain intensity (−1.5 [95% CI, −2.2 to −0.7] points; *P* < .001) and mean RMDQ (−2.8 [95% CI, −4.3 to −1.3] points; *P* < .001) scores. At 24 weeks, the improvements in pain (mean change, −2.3 [95% CI, −3.1 to −1.6] points; *P* < .001) and RMDQ (mean change, −4.6 [95% CI, −6.1 to −3.1] points; *P* < .001) scores were sustained. At 12 weeks, yoga now participants reported 21.4 (95% CI, 5.2-37.6) absolute percentage points less use of any analgesic medication during the past week than yoga later participants and at 24 weeks, 21.2 absolute percentage points (95% CI, 5.2%-37.3%). Improvements in sleep quality were greater for participants in yoga now than among participants in yoga later at 12 weeks (mean change, 0.4 [95% CI, 0.1-0.7] points; *P* = .008) and 24 weeks (mean change, 0.4 [95% CI, 0.1-0.7] points; *P* = .005).

**Conclusions and Relevance:**

Results of this randomized clinical trial of 140 adults with CLBP who were members of a large health system self-insured health plan suggest that virtual yoga classes may be a feasible, safe, and effective treatment option.

**Trial Registration:**

ClinicalTrials.gov Identifier: NCT05319691

## Key Points

**Question:**

What is the effectiveness of virtually delivered yoga classes for chronic low back pain in a health system employee population?

**Findings:**

In this randomized clinical trial, 140 participants with chronic low back pain were randomized to participate in a 12-week virtual group yoga class or a wait-list control. After the intervention, participants in yoga had significantly greater improvements than participants in the wait-list control group in back pain intensity and back-related function with no serious adverse effects.

**Meaning:**

Findings of this study indicate that a 12-week therapeutic virtual yoga program for chronic low back pain is feasible, safe, and effective.

## Teaser

This randomized clinical trial examined whether participating in virtual live streamed yoga is an effective treatment option compared with being on a wait-list among adults with chronic low back pain.

## Introduction

Low back pain (LBP) is the leading cause of disability and health care costs in the United States and a highly prevalent condition faced by health care employees.^1,2,3,4^ Lifetime prevalence of LBP among health care employees is approximately 55%, with workplace body mechanics and posture, work-related psychosocial stressors, and lack of physical activity as primary risk factors for the development of LBP.^4,5,6^ Despite a national trend for increased insurance spending on LBP, worsening back-related outcomes have been observed at the population level.^7^ Medications, injections, and surgery are widely used, but are costly with possible adverse effects and variable effectiveness.^8,9,10,11,12,13^ Despite the availability of these treatments, at least one-third of patients visiting a primary care clinician for LBP report ongoing pain and disability up to 1 year later.^14,15,16^ Furthermore, among patients with acute LBP, the incidence of high-impact chronic pain has been estimated to be between 15% and 30%.^17^ This finding helps explain the substantial emotional, physical, and financial burden that LBP causes employees, their families, society, health care systems, and payers. Thus, there is a clear need to study promising therapies that may be helpful adjuncts or alternatives for LBP that are also low-cost, safe, and accessible.

Clinical guidelines for chronic LBP (CLBP) from the American College of Physicians^18^ and the US Department of Health and Human Services Pain Management Best Practices Inter-Agency Task Force^19^ recommend a stepped-care approach for the treatment of CLBP, starting with nonpharmacological approaches, including yoga and physical therapy, followed by nonopioid medications and then opioid medications, only if benefits clearly outweigh the risks. Therapeutic yoga is supported as an evidence-based treatment for CLBP^20^; numerous randomized controlled trials (RCTs), systematic reviews, and meta-analyses have demonstrated its efficacy in improving pain intensity and back-related function.^21,22,23,24,25,26,27,28,29^ Yoga has also been shown to be noninferior to physical therapy for chronic LBP.^25^ Despite these benefits, decisions to recommend yoga to patients is highly reliant on trained instructors, availability of CLBP-specific yoga programs, clinician awareness of the evidence, and patient and health care professional preferences.^28^

It is uncommon for patients with CLBP to receive therapeutic yoga in health care systems or through their insurance plan. Attending yoga in person is a significant barrier for some individuals.^30^ This is especially relevant for individuals working in health care due to scheduling demands associated with patient care, in addition to personal demands outside of work hours. As a result, medications and other higher-risk interventions are often prescribed, despite their limited effectiveness and risk of adverse effects.^8,9,10,11,13,31^ It is important to increase access to evidence-based nonpharmacologic practices such as yoga within health care settings to decrease the burden of LBP. Delivering yoga virtually also has the potential to address barriers to accessing this intervention, including scheduling, travel, mobility challenges, and some patients not wanting to be seen by others when engaging in yoga. Prior studies have found that virtually delivered yoga is effective and feasible to implement for stress reduction among health care workers during the COVD-19 pandemic,^32^ patients with irritable bowel syndrome,^33^ and patients with chemotherapy-induced peripheral neuropathy pain.^34^ To our knowledge, no studies to date have evaluated whether the effectiveness of in-person yoga for CLBP is translatable to a virtual platform. If it is, this finding will have significance for implementation of yoga for CLBP into treatment pathways. Given this gap in knowledge, we conducted an RCT with members of Cleveland Clinic’s self-insured Employee Health Plan (EHP), with the main emphasis being intervention effectiveness in a novel population and a new delivery method (virtual). Here, we present data from the embedded 24-week single-blinded (investigator), 2-arm RCT of virtual live streamed yoga (yoga now) and a wait-list control (yoga later).

## Methods

### Participants

We recruited Cleveland Clinic EHP members from Northeast Ohio and Florida between May 3 and October 31, 2022. The EHP office generated a list of 501 potential participants with a billing diagnosis of CLBP (*International Statistical Classification of Diseases, Tenth Revision*, code M54.5) in the last 3 years and mailed them a recruitment letter. Flyers were also posted within employee areas of the hospital, advertisements were posted on the intranet homepage, and emails were sent to employees at 1 Cleveland Clinic Ohio hospital location. Sample size was determined by power analysis to detect minimal clinically important differences between groups in pain intensity and back-related function. Inclusion criteria were (1) adults 18 to 64 years of age, (2) current nonspecific LBP persisting at least 12 weeks, (3) mean LBP intensity of 4 or higher for the previous week on an 11-point numeric rating scale (0-10, with higher numbers indicating worse pain), (4) daily back pain interference half or more than half of the days in the past 6 months,^35^ (5) English language comprehension, and (6) insurance provided by the Cleveland Clinic Employee Health Plan, which covers employees, their spouse, and children less than 26 years of age. Exclusion criteria included any severe psychiatric or medical comorbidity that would make study participation unsafe or not feasible, at the discretion of the principal investigator (R.S.), who is a practicing clinician. The study was approved by the Cleveland Clinic Institutional Review Board and registered with ClinicalTrials.gov prior to recruitment. Oral informed patient consent was provided during the telephone interview; written consent was not provided because the study was deemed minimal risk by the Cleveland Clinic Institutional Review Board. The Trial Protocol is provided in Supplement 1. This study report follows the 2017 Consolidated Standards of Reporting Trials (CONSORT) reporting guideline for randomized trials assessing nonpharmacologic treatments.

### Procedure

Recruitment started May 3, 2022, and ended October 31, 2022. Interested EHP members contacted the study team, provided remote informed consent, and were screened via a standardized telephone questionnaire. Eligible individuals were provided with detailed information about the study. Participants were emailed a link to complete a baseline questionnaire via REDCap. The questionnaire included a question about race in which participants could choose from American Indian or Alaska Native, Asian, Black, White, or Multiple races or decline to answer and a question about ethnicity, with choices of Hispanic and not Hispanic. Race and ethnicity categories were sourced from 2022 National Institutes of Health guidelines. Race and ethnicity were assessed in this study to more fully characterize the participants. Participants were then randomly assigned by the biostatistician (B.H.), who was blinded to condition assignment, using R (R Project for Statistical Computing) in a 1:1 ratio to either yoga now (intervention) or yoga later (wait-list control) groups. Yoga now included weekly virtual therapeutic yoga sessions for 12 weeks followed by a 12-week follow-up assessment period. For yoga later, participants were offered nonstudy yoga classes after 24 weeks. The investigators were blinded to treatment assignment until dataset lock; due to the nature of the intervention, participants and study staff were not blinded to treatment assignment. Study staff enrolled participants and informed them of their treatment assignment. Yoga interventions started July 1, 2022, and data collection was completed June 30, 2023.

### Yoga Intervention

Yoga instructors delivered a manualized and reproducible hatha yoga intervention targeting the yoga-naïve individual and designed to maximize effectiveness and safety in a population with CLBP. The intervention and instructor manuals (Supplement 3) were adapted for virtual delivery by our yoga instructor team from 3 previous studies: an expert panel-designed protocol tested in a pilot study of 30 participants with CLBP,^36^ dosing study of 95 participants,^37^ and larger noninferiority trial of 320 participants.^25^ We delivered the yoga sessions over Webex, a Health Insurance Portability and Accountability Act–secure virtual platform. Each class was 60 minutes in length and followed the same weekly format (eTable 1 in Supplement 2). Participants gradually learned a sequence of 12 to 15 yoga poses, and the degree of difficulty increased each class. The yoga protocol provided variations and used aids (eg, chair, strap) to accommodate a range of physical abilities. Participants were strongly encouraged to practice at home for 30 minutes daily outside of class. To facilitate at-home practice, participants were provided with a yoga mat, a participant manual (Supplement 3), and access to prerecorded yoga classes corresponding to the session content each week. A study team member (D.G., J.B., R.W., H.W., P.T., J.G.) virtually observed and assessed 10% of online yoga classes for instructor fidelity to the protocol using a checklist.

Six yoga instructors who completed at least 200 hours of yoga teacher training and were registered by Yoga Alliance (including J.B., R.W., H.W., P.T., J.G.) were available to teach 8 cohorts of weekly yoga classes. All instructors had prior experience teaching yoga virtually. Cohorts were assembled and started on a rolling basis as the study progressed. Two yoga instructors were present for each class: one taught and the other observed for safety, providing feedback to participants to correct alignment and reduce risk of harm. Each class had a planned cohort size of 8 to 10.

### Control Group

Yoga later participants completed the same assessments as yoga now participants. During the 24-week study period, participants were encouraged to continue their usual medical care but discouraged from beginning a new yoga practice. Following the final assessment point, all participants were offered 12 weekly yoga classes with no formal assessments.

### Measures

All self-report measures were completed remotely through REDCap. Baseline measures were administered within 1 week prior to randomization. Participants were reassessed at 6 weeks (intervention midpoint), 12 weeks (immediately after the intervention), and 24 weeks (12 weeks postintervention). Coprimary outcomes were pain intensity (assessed by an 11-point numeric rating scale: 0 = no pain and 10 = worst possible pain^38^) and back-related function (assessed by the 23-item modified Roland Morris Disability Questionnaire [RMDQ], scores ranged from 0-23, with higher scores reflecting poorer function^39,40^) at 12 weeks. Additional outcomes included prescription and over-the-counter analgesic medication use during the past 7 days (yes or no) and sleep quality (Patient-Reported Outcomes Measurement Information System [PROMIS] Sleep Disturbance Short Form 8a, item 1; score range, 0-4, with higher scores reflecting better sleep quality). We conducted a post hoc exploratory analysis of the proportion of participants in each group achieving a clinically important change in their primary outcomes (≥30% reduction from baseline). We tracked in-class attendance, and at-home yoga practice was tracked with an electronic weekly yoga diary, which noted the number of minutes practiced outside of class each day. Adverse events were collected through questionnaires and direct reports to study staff. We also collected data (eg, interviews with key stakeholders) to inform implementation for health care employees, which will be reported separately.

### Statistical Analysis

The sample size calculation assumed a conservative 20% attrition rate for the intervention group and no change in the control group. We assumed a minimal clinically significant decrease in pain intensity as 2.0 points^41^ and RMDQ score as 3.0 points.^42^ A sample size of 140 participants was estimated to provide 99% and 82% statistically significant differences in pain intensity and back-related function, respectively. To account for the 2 coprimary outcomes, the α-level was set to 0.025. Randomization occurred after administering baseline surveys using a computerized randomization procedure built into REDCap. Participants were randomized using a 1:1 ratio to yoga now or yoga later groups. We used permuted variably sized block randomization with block sizes of 6, 12, and 18.

The statistical analysis followed the intention-to-treat principle. For analysis of outcomes, linear mixed-effects models were used, which included time, group, their interaction as fixed effects, and a Gaussian random intercept at the participant level to account for the clustered data structure. Analyses of mean changes between groups included the baseline score as a covariate. The primary comparison was change from baseline to week 12 between the 2 groups, and the Bonferroni approach was used to account for the coprimary outcomes to determine statistical significance (ie, a 2-sided *P* < .025). The linear mixed-effect models assumed data to be missing at random. A sensitivity analysis was conducted using multiple imputation to address missing data. To impute the missing data of the primary outcomes, the chained equation approach was applied to the study dataset with baseline variables, pain intensities, and RMDQ scores arranged chronologically. The results across 5 imputed datasets were summarized according to the Rubin rule. All analyses were conducted in SAS, version 9.4 (SAS Institute Inc), and RStudio (R Project for Statistical Computing).

## Results

### Participants

**Figure 1.**
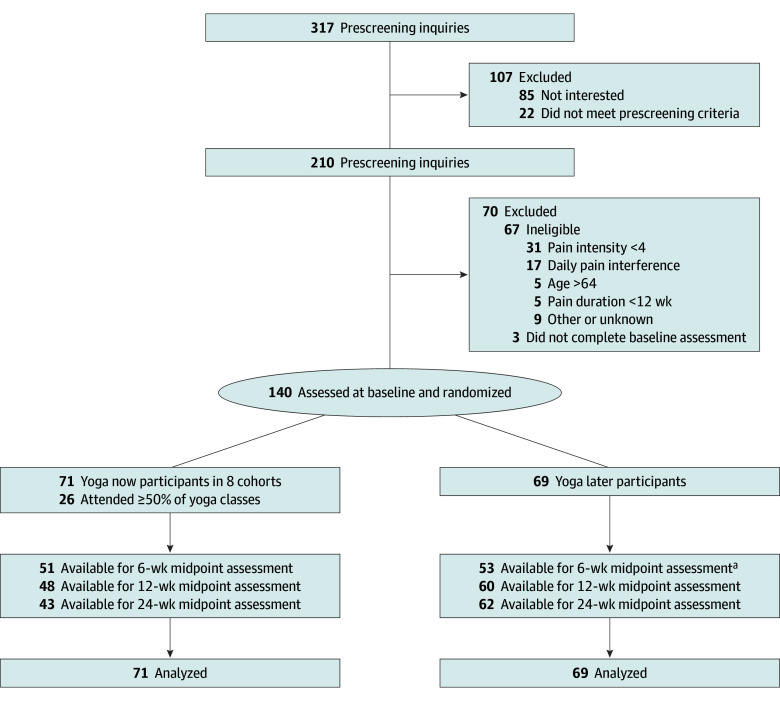
Study Flow Diagram ^a^Due to an error in study coordination and survey administration, only 54 of 69 participants in the yoga later group received the 6-week assessment. Of 54 participants, 53 completed the assessment.

We enrolled 140 participants (yoga now = 71; yoga later = 69), who were primarily female (113 [80.7%]; 27 [19.3%] male), middle-aged (mean [SD] age, 47.8 [11.7] years; range, 38-59 years), and college-educated (103 [73.5%]); 17 participants (12.1%) identified as Black, 115 (82.1%) as White, and 7 (5.0%) as other (including American Indian or Alaska Native, Asian, or multiracial; groups were combined to prevent participant identification), and 1 (0.7%) declined to answer; 8 participants (5.7%) self-identified as Hispanic, and 132 (94.3%) as not Hispanic (Figure 1, Table 1). Mean (SD) baseline pain intensity (5.7 [1.5]) and RMDQ (12.1 [4.4]) scores reflected moderate back pain and back function impairment. At baseline, 104 participants (74.3%) used any form of analgesic medication during the past 7 days, including nonsteroidal anti-inflammatory drugs ([NSAIDs] 90 [64.3%]), acetaminophen (46 [32.9%]), muscle relaxants (23 [16.4%]), opioids (7 [5.0%]), topicals (10 [7.1%]), and other medications (31 [22.1%]). Sleep quality was on average fair to poor.

**Table 1.** Participant Characteristics

Factor	Participants, No. (%)		
	Total (N = 140)	Yoga now (n = 71)	Yoga later (n = 69)
Sex			
Female	113 (80.7)	57 (80.3)	56 (81.2)
Male	27 (19.3)	14 (19.7)	13 (18.8)
Age, mean (SD), y	47.8 (11.7)	48.9 (11.2)	46.7 (12.1)
Race			
Black	17 (12.1)	7 (9.9)	10 (14.5)
White	115 (82.1)	61 (85.9)	54 (78.3)
Other^a^	7 (5.0)	3 (4.2)	4 (5.7)
Declined to answer	1 (0.7)	0	1 (1.4)
Ethnicity			
Hispanic	8 (5.7)	4 (5.6)	4 (5.8)
Not Hispanic	132 (94.3)	67 (94.4)	65 (94.2)
Education			
High school	7 (5.0)	2 (2.8)	5 (7.2)
Some college	30 (21.4)	17 (23.9)	13 (18.8)
College	65 (46.4)	35 (49.3)	30 (43.5)
Graduate school	38 (27.1)	17 (23.9)	21 (30.4)
Employment			
Working	135 (96.4)	69 (97.2)	66 (95.7)
Disabled back pain	2 (1.4)	1 (1.4)	1 (1.4)
Caregiver	1 (0.7)	1 (1.4)	0
Full time volunteer	1 (0.7)	0	1 (1.4)
Retired	1 (0.7)	0	1 (1.4)
Pain intensity score, mean (SD)^b^	5.7 (1.5)	5.7 (1.5)	5.8 (1.6)
RMDQ score, mean (SD)^c^	12.1 (4.4)	11.7 (4.5)	12.4 (4.3)
BMI, mean (SD)	29.2 (7.2)	29.2 (6.4)	29.2 (7.9)
Smoking			
Never	100 (71.4)	50 (70.4)	50 (72.5)
Former	37 (26.4)	19 (26.8)	18 (26.1)
Current	2 (1.4)	2 (2.8)	0
Declined to answer	1 (0.7)	0	1 (1.4)
Pain history duration			
>5 y	73 (52.1)	36 (50.7)	37 (53.6)
1-5 y	59 (42.1)	29 (40.8)	30 (43.5)
<1 y	8 (5.7)	6 (8.5)	2 (2.9)
History of epidural injection			
No	82 (58.6)	45 (63.4)	37 (53.6)
1-3	35 (25.0)	12 (16.9)	23 (33.3)
>3	23 (16.4)	14 (19.7)	9 (13.0)
History of low back surgery			
No	120 (85.7)	59 (83.1)	61 (88.4)
1	14 (10.0)	9 (12.7)	5 (7.2)
>1	6 (4.3)	3 (4.2)	3 (4.3)
Sciatica^d^			
No	57 (40.7)	32 (45.1)	25 (36.2)
Not sure	10 (7.1)	7 (9.9)	3 (4.3)
Yes	73 (52.1)	32 (45.1)	41 (59.4)
Pain medication use in previous week			
Any pain medication	104 (74.3)	49 (71.0)	55 (77.5)
NSAID	90 (64.3)	39 (56.5)	51 (71.8)
Acetaminophen	46 (32.9)	21 (30.4)	25 (35.2)
Muscle relaxant	23 (16.4)	12 (17.4)	11 (15.5)
Opioid	7 (5.0)	3 (4.3)	4 (5.6)
Topical cream	10 (7.1)	6 (8.7)	4 (5.6)
Other	31 (22.1)	15 (21.7)	16 (22.5)
Sleep quality score, mean (SD)^e^	1.8 (0.8)	1.9 (0.1)	1.7 (0.1)

Abbreviations: BMI, body mass index (calculated as weight in kilograms divided by height in meters squared); NSAID, nonsteroidal anti-inflammatory drug; RMDQ, Roland Morris Disability Questionnaire.

^a^ includes American Indian or Alaska Native, Asian, and multiracial.

^b^ Measured using an 11-point numerical rating scale for mean pain intensity in the previous week, with 0 indicating no pain and 10 indicating worst pain possible.

^c^ Measure of back-related function with scores ranging from 0 to 23, with higher scores representing poorer function.

^d^ Whether back pain has spread down 1 or both legs in the past 2 weeks.

^e^ Measured using the PROMIS Sleep Disturbance Short Form 8a, item 1. Scores range from 0 to 4, with higher scores reflecting better sleep quality.

Yoga classes had between 0 and 9 participants per class (median [IQR], 3 [2-5]). Participants assigned to yoga now had an adherence rate (≥50% class attendance) of 36.6% and attended a median (IQR) of 4 (3-6) classes. For 65 participants who returned home practice logs, participants practiced an estimated median (IQR) of 4 (3-6) days per week, and a mean (SD) of 28.1 (15.6) minutes per day. Follow-up assessment completion rates were lower in the yoga now group than in the yoga later group at 6 weeks (71.8% vs 76.8%), 12 weeks (67.6% vs 87.0%), and 24 weeks (60.6% vs 89.9%).

### Primary Outcomes

**Figure 2.**
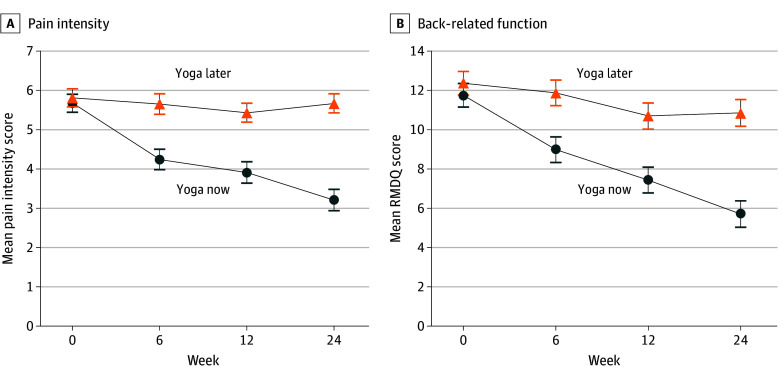
Pain Intensity and Back-Related Function From Baseline to 24 Weeks A, Pain intensity was measured using an 11-point numerical rating scale for mean pain intensity in the previous week, with 0 indicating no pain and 10 indicating worst pain possible. Error bars represent SE. B, Back-related function was measured using the Roland Morris Disability Questionnaire (RMDQ), a measure of back-related function with scores ranging from 0 to 23, with higher scores representing poorer function. Error bars represent SE.

Figure 2 shows the progression of pain intensity and RMDQ scores from baseline to 24 weeks. Table 2 and Table 3 show mean changes in pain intensity and RMDQ scores from baseline to 6, 12, and 24 weeks and between-group mean change differences (yoga now vs yoga later) at each time point. Compared with participants in yoga later, participants in yoga now showed significantly greater (*P* < .001) mean reductions in pain intensity scores from baseline at 6 weeks (mean change, −1.3 [95% CI, −2.1 to −0.6] points) and 12 weeks (mean change, −1.5 [95% CI, −2.2 to −0.7] points). Similar results were observed for back-related function: participants in the yoga now group showed significantly greater mean reductions in RMDQ scores from baseline to 6 weeks (mean change, −2.4 [95% CI, −3.9 to −0.9] points) and 12 weeks (mean change, −2.8, [95% CI, −4.3 to −1.3] points). Primary outcome results using multiple imputation demonstrated similar results (eTable 2 in Supplement 2).

**Table 2.** Primary and Secondary Outcomes

Outcome Measure	Mean change from baseline (95% CI)					
	6 wk	*P* value	12 wk	*P* value	24 wk	*P* value
**Pain intensity^a^**						
Yoga now	−1.4 (−1.99 to −0.89)	NA	−1.8 (−2.3 to −1.2)	NA	−2.4 (−3.0 to −1.9)	NA
Yoga later	−0.1 (−0.6 to 0.4)	NA	−0.3 (−0.8 to 0.2)	NA	−0.1 (−0.6 to 0.5)	NA
Between-group mean difference	−1.3 (−2.1 to −0.6)	<.001	−1.5 (−2.2 to −0.7)	<.001	−2.3 (−3.1 to −1.6)	<.001
**Back-related function (RMDQ)^b^**						
Yoga now	−2.7 (−3.8 to −1.6)	NA	−4.3 (−5.5 to −3.2)	NA	−6.0 (−7.1 to −4.9)	NA
Yoga later	−0.3 (−1.4 to 0.7)	NA	−1.6 (−2.6 to −0.6)	NA	−1.4 (−2.4 to −0.4)	NA
Between-group mean difference	−2.4 (−3.9 to −0.9)	.002	−2.8 (−4.3 to −1.3)	<.001	−4.6 (−6.1 to −3.1)	<.001
**Sleep quality^c^**						
Yoga now	0.3 (0.1 to 0.5)	NA	0.4 (0.2 to 0.7)	NA	0.5 (0.3 to 0.7)	NA
Yoga later	0.1 (−0.1 to 0.3)	NA	0.04 (−0.2 to 0.2)	NA	0.1 (−0.2 to 0.3)	NA
Between-group mean difference	0.2 (−0.1 to 0.5)	.17	0.4 (0.1 to 0.7)	.008	0.4 (0.1 to 0.7)	.005

Abbreviation: NA, not applicable; RMDQ, Roland Morris Disability Questionnaire.

^a^ Measured using an 11-point numerical rating scale for mean pain intensity in the previous week, with 0 indicating no pain and 10 indicating worst pain possible.

^b^ Scores range from 0 to 23, with higher scores representing poorer function.

^c^ Measured using the PROMIS Sleep Disturbance Short Form 8a, item 1. Scores range from 0 to 4, with higher scores reflecting better sleep quality.

**Table 3.** Pain Medication Use at 12 and 24 Weeks in Yoga Now vs Yoga Later Participants

Pain medication use in previous week	12 wk				24 wk			
	Yoga now participants, No. (%)	Yoga later participants, No. (%)	Absolute difference, % (95% CI)	*P* value	Yoga now participants, No. (%)	Yoga later participants, No. (%)	Absolute difference, % (95% CI)	*P* value
Any pain medication	27 (38.0)	41 (59.4)	21.4 (5.2 to 37.6)	.04	23 (32.4)	37 (53.6)	21.2 (5.2 to 37.3)	.01
NSAID	20 (28.2)	34 (49.3)	21.1 (5.3 to 36.9)	.01	12 (16.9)	24 (34.8)	17.9 (3.7 to 32.1)	.02
Acetaminophen	11 (15.5)	13 (18.8)	3.3 (−9.1 to 15.8)	.60	11 (15.5)	17 (24.6)	9.1 (−4.1 to 22.3)	.18
Muscle relaxant	3 (4.2)	9 (13.0)	8.8 (−4.0 to 18.0)	.06	3 (4.2)	11 (15.9)	11.7 (1.9 to 21.5)	.02
Opioid	2 (2.8)	2 (2.9)	0.1 (−5.4 to 5.6)	.98	1 (1.4)	4 (5.8)	4.4 (−1.8 to 10.5)	.16
Topical cream	1 (1.4)	2 (2.9)	1.5 (−3.3 to 6.3)	.54	1 (1.4)	2 (2.9)	1.5 (−3.3 to 6.3)	.54
Other	5 (7.0)	12 (17.4)	10.3 (−0.4 to 21.1)	.06	5 (7.0)	9 (13.0)	6.0 (−3.9 to 15.9)	.24

Abbreviation: NSAID, nonsteroidal anti-inflammatory drug.

### Secondary and Exploratory Outcomes

Table 2 and Table 3 also summarize secondary and exploratory outcomes. From baseline to 24 weeks, participants in yoga now showed significantly greater reductions in pain intensity (mean change, −2.3 [95% CI, −3.1 to −1.6] points; *P* < .001) and RMDQ scores (mean change, −4.6 [95% CI, −6.1 to −3.1] points; *P* < .001) than yoga later participants (Table 2). The percentages of participants experiencing clinically important changes in pain intensity (38.0% vs 20.3%) and RMDQ (42.3% vs 24.6%) scores at 12 weeks were approximately 1.7- to 1.9-fold greater for yoga now compared with yoga later (eTable 3 in Supplement 2). At 12 and 24 weeks, yoga now participants reported 21.2 absolute percentage points (95% CI, 5.2%-37.3%) less use of any analgesic medication during the past week than yoga later participants (Table 3). The most significant reductions were observed with NSAIDs, with 17.9 absolute percentage points (95% CI, 3.7%-32.1%). Yoga now participants compared with yoga later participants showed significantly greater improvements in sleep quality scores from baseline to 12 weeks (mean change, 0.4 [95% CI, 0.1-0.7] points; *P* = .008) and to 24 weeks (mean change, 0.4 [95% CI, 0.1-0.7] points; *P* = .005) (Table 2).

### Adverse Events

Adverse events were uncommon in both groups. In yoga now, 3 participants reported transient exacerbation of back pain possibly related to the intervention. One participant in yoga later reported a flare-up of pre-existing neck pain.

## Discussion

In our 12-week RCT of 140 adults with CLBP, participants receiving 12 weeks of virtual live streamed yoga classes experienced clinical and statistically significant improvements in pain intensity and back-related function compared with participants on a wait-list. Yoga now participants also reported reduced pain medication use and improved sleep quality. Improvements in the yoga now group were maintained at 24 weeks. Adverse events were uncommon and not serious. Given the demonstrated noninferiority of yoga to physical therapy,^25^ structured virtual yoga programs and physical therapy are reasonable choices for patients with CLBP depending on accessibility, cost, and patient preference. These findings support the call by the National Academy of Medicine for increased evidenced-based pain treatments that can be disseminated via technology-based platforms.^43^

The improvements in pain intensity and back-related function align with prior RCTs of in-person therapeutic yoga classes for CLBP,^21,22,23,24^ with our sample experiencing even greater improvements at 12 weeks (postintervention) compared with prior trials. This finding is notable, considering only 36.6.% of yoga now participants attended at least 50% of classes. There are 2 plausible explanations for our low adherence rate not impacting the effectiveness of yoga. First is the availability of recorded classes for participants to use for practice or to watch at a later date if they missed class. This is a flexible option not always available for in-person trials and has potential to enhance the feasibility, acceptability, and effectiveness of yoga for CLBP. Second, participants engaged in regular home yoga practice (4 days/week; 28.1 minutes/d), regardless of whether they attended live classes. Together, these 2 explanations justify future trials to test dosage of virtual live streamed yoga classes. Although the majority of yoga studies for CLBP have used a 12-week course sequence,^44^ offering fewer live classes (eg, 6 weekly classes) with making recordings available and encouraging home practice may yield results similar to 12 live streamed weekly classes.

Our trial enrolled a sample with similar demographics to many prior yoga trials for CLBP,^21,22,23,24^ namely participants who are mostly middle-aged, White, non-Hispanic, college-educated women. However, given documented racial disparities in pain and disability,^43,45^ we understand the pressing need to target a more diverse study sample in future trials by addressing barriers faced by understudied and minoritized communities.^25^ Finally, we observed similar baseline characteristics reported in prior yoga trials, including moderate levels of pain and disability.^25,46^ However, the proportion of our sample reporting baseline opioid use was approximately 4-fold less compared with participants in prior yoga trials for CLBP.^25,46^ This result likely reflects efforts in recent years to decrease opioid prescriptions within the Cleveland Clinic and nationally. Due to the COVID-19 pandemic, a rapid pivot to online yoga classes occurred nationally without knowledge of the effectiveness of virtual yoga delivery. As such, our findings add to the existing literature on the effectiveness of virtual mindfulness interventions in chronic pain.^47,48^ The effect sizes (absolute between-group mean differences) observed in our sample from baseline to 12 weeks for pain (1.5) and disability (2.8) are larger than prior yoga trials for CLBP that used an educational book for control (eg, 0.3 and 1.3, respectively;^25^ 0.2 and 1.0, respectiviely^46^). This finding may be explained by a modest to small effect size for education compared with our use of a wait-list control, which showed stability in pain severity and back function throughout the trial. By labeling our groups yoga now and yoga later, we were able to maintain good engagement of yoga later participants, thereby lowering the risk of drop out.

### Strengths and Limitations

Strengths of this study were the use of a randomized design with a wait-list control group. We also examined the effects of yoga on sleep quality in patients with CLBP, an important health construct that is rarely assessed in yoga-for-back-pain RCTs but is a core patient-reported outcome domain in pain clinical trials.^49,50^ Finally, we created and used a manualized and reproducible hatha yoga intervention designed for the yoga-naïve individual to maximize generalizability, effectiveness, and safety in a CLBP population.

Study limitations included disproportionately more missing data in yoga now compared with yoga later and low rates of class attendance. The latter finding may be explained in part by the nature of the trial with no financial incentives for participation and only modest effort by the research team to promote attendance and data collection through email reminders. Second, we were unable to mask participants to treatment assignment and used only patient-reported outcome measures.^51^ Accurate assessment of home yoga practice is limited due to potential self-report bias and incomplete reporting. More reliable methods are needed to measure home practice and determine its importance. Additionally, we did not include a longer-term follow-up assessment (eg, 1 year).

## Conclusions

This RCT found that in a sample of adult patients with CLBP who were members of a large health care system self-insured health plan, 12 weeks of virtually delivered group yoga classes (compared with a wait-list control) led to significantly greater improvements in back pain intensity and back-related function, with maintenance of improvement at 24-weeks’ follow-up and no serious adverse events. Through reducing barriers to in-person participation, virtual yoga classes may be a feasible, safe, and effective treatment option for CLBP. Future work should include scaling virtual therapeutic yoga within health care systems, assessing longer-term follow-up and cost-effectiveness, comparing 6 vs 12 weeks of classes, and testing recruitment strategies to increase the diversity of participants.

## Notes

Supplement 1.
Trial Protocol

Supplement 2. **eTable 1.** Yoga Class Format **eTable 2.** Sensitivity Analysis of Primary Outcomes Using Multiple Imputation **eTable 3.** Participants Experiencing Clinically Important Changes in Co-Primary Outcomes (30% or Greater Reduction From Baseline)

Supplement 3.
Guidebook/Manual

Supplement 4.
Data Sharing Statement
