# Supplementary material for: Effectiveness of Virtual Yoga for Chronic Low Back Pain: A Randomized Clinical Trial
Source: JAMA Netw Open. 2024 Nov 1;7(11):e2442339. doi: 10.1001/jamanetworkopen.2024.42339 (PMC11530940; doi:10.1001/jamanetworkopen.2024.42339)
Supplement: Supplement 1. — Trial Protocol [file jamanetwopen-e2442339-s001.pdf]

Trial Protocol

TITLE: YOGA FOR CHRONIC LOW BACK PAIN IN EHP

RUNNING/ABBREVIATED TITLE: YOGA FOR cLBP IN EHP

Principal Investigator (P.I.):
Robert B. Saper, M.D., M.P.H.
Chair, Department of Wellness & Preventive Medicine
Primary Care Institute
saperr@ccf.org
216-448-4325

STUDY OVERVIEW

This study uses clinical trial and implementation science methodology to specifically assess the clinical effectiveness and implementation of yoga into the management of chronic low back pain (cLBP) within the Cleveland Clinic (CC) Employee Health Plan (EHP). We will use a Type 1 hybrid effectiveness-implementation study design,[23] which tests the effectiveness of a clinical intervention while collecting data on implementation.

Specific Aims:

Aim 1: Conduct a randomized controlled trial to determine the clinical effectiveness of a 12-week virtual yoga intervention (Yoga Now) compared to a wait-list control group (Yoga Later) for cLBP in the CC Employee Health Plan population. We hypothesize that at 12 weeks the Yoga Now group will have clinically and statistically significant improvements in pain and function compared to the Yoga Later group.

Co-primary outcomes will be self-reported average pain intensity for the previous week (11-point numerical rating scale ranging from 0=no pain to 10=worst possible pain) and back-related function (modified Roland Morris Disability Questionnaire ranging from 0=no functional impairment to 23=worst impairment of function). *Secondary outcomes* will include analgesic use, global improvement, and overall satisfaction. Yoga participants will receive a reproducible standardized weekly yoga intervention delivered virtually with additional resources for home practice. The wait-list control participants will receive usual back pain care. Data collection will occur at baseline, 6 weeks, 12 weeks, and 24 weeks. The primary time point of interest will be 12 weeks. After the 24-week study period the wait-list control group will be offered the same protocol but in a non-study format without additional data collection.

Aim 2: Use mixed methods to measure implementation outcomes and assess facilitators and barriers to yoga's implementation in the CC EHP setting. We hypothesize that implementation of yoga in the context of a RCT in CC EHP will be successful and facilitated by the partnership with EHP for recruitment and virtual intervention.

Study record tracking will be used to measure feasibility (e.g., rate of recruitment, loss-to-follow up) and intervention costs (e.g., yoga instructors, yoga mats). Study staff observation of classes using a checklist will assess fidelity of the intervention to the standardized yoga protocol. Acceptability, appropriateness, and adoption of yoga for cLBP will be assessed through quantitative measures (satisfaction, home practice logs) and open-ended questions to participants in surveys (Yoga Now Questionnaire Supplements, Weeks 12 and 24).

Aim 3 (Exploratory): Demonstrate the feasibility of evaluating cost-effectiveness of yoga compared to usual care for cLBP from the perspective of the payer.

Cost of implementing the intervention will be measured. Total and back-related medical expenditures for the six-month study period will be compared between the yoga and wait-list control groups. We hypothesize that total and back-related medical expenditures by CC EHP for the yoga group will be less than the usual care group.

BACKGROUND

Low back pain (LBP) is common with enormous impact on patient suffering, healthcare cost, and provider burden.[1] Up to 80% of U.S. adults will experience at least one episode of LBP in their lifetime.[2] Chronic LBP affects roughly 10% of U.S. adults.[2] The prevalence of LBP globally has increased from 377.5 million in 1990 to 577.0 million in 2017, and is the leading cause of disability worldwide.[26, 27, 28] LBP is the second most common reason for doctor visits.[27,1] The morbidity and cost of LBP, especially chronic LBP, is particularly high due to comorbid pain (e.g., neck pain, osteoarthritis, fibromyalgia, headache) and mental health conditions (e.g., depression, anxiety, PTSD). Back pain is a financial burden on society, families, and individuals. Back and neck pain in 2016 ranked highest in U.S. health expenditures ($134.5 billion) - higher than diabetes, ischemic heart disease, and depression.[3] Patients and their families bore 9.2% ($12.4 billion) of these costs. Despite a national trend for increased spending on LBP, worsening outcomes have been observed at the population level, including increasing levels of disability.[4]

Treatment of LBP. A clear gap has been identified between the evidence base and real world treatment of LBP nationally and at CC. Clinical guidelines from the American College of Physicians (ACP) issued in 2017 for acute and chronic LBP recommend non-pharmacologic approaches should be used first, non-opioid medications second, and thereafter opioid medications only if benefits clearly outweigh the risks.[5] ACP guidelines recommend acupuncture, massage, spinal manipulation, and superficial heat for acute LBP. For cLBP, yoga, spinal manipulation, exercise (PT), acupuncture, massage, mindfulness, cognitive behavioral therapy (CBT), and others are recommended. This stepped care approach is based on AHRQ systematic reviews[6] and supported by guidelines from the HHS Pain Management Best Practices Inter-Agency Task Force.[31] Meta-analyses of trials utilizing yoga and mindfulness therapies also demonstrate potential to reduce opioid use.[7] Economic evaluations suggest group interventions such as yoga can be cost saving.[8]

Despite the evidence and guidelines, it is unusual for cLBP patients to receive recommended non-pharmacologic treatments in health care systems, including CC. Causes are multiple and include lack of reimbursement, licensed providers, awareness, and adequate time for busy clinicians to counsel patients about options and shared decision making. As a result, medications and in particular opioids are often prescribed, despite minimal evidence for their effectiveness.[9] Back pain is the most common non-cancer diagnosis associated with opioid prescribing.[29] Pain is a major upstream driver of opioid use disorder, overdose, and death. Almost 5% of individuals receiving opioid prescriptions go on to misuse or addiction, with the most commonly reported motivation for opioid misuse being the relief of pain.[11] Almost half of persons who use heroin and/or fentanyl report their addiction began with prescription opioids.

Thus, studying and implementing evidence-based non-pharmacologic interventions is an important strategy for improving pain management and reducing opioid use disorder.

STUDY DESIGN & METHODS

We will use a single-blinded (investigator) two-arm randomized control trial (RCT) design. The trial will be 24 weeks long and divided into two distinct parts: an initial 12-week Treatment Phase followed by a 12-week Follow-up Phase. Participants will be randomized 1:1 into weekly virtual yoga classes (***Yoga Now***) or a wait-list control (***Yoga Later***) who will receive usual care. After the 24-week study period, participants in Yoga Later will be offered the yoga intervention in a non-study format.

Study Participants:

Participants will be recruited from members of the Cleveland Clinic Employee Health Program (CC EHP). There are approximately 105,000 members, including caregivers and their families, across Northeast Ohio, Florida, and Las Vegas.

Inclusion criteria:

- 18-64 years old
- Current non-specific LBP persisting ≥12 weeks with average pain intensity ≥4 for the previous week on an 11-point numerical rating scale
- Ability to speak and understand English
- Agrees to be visualized by yoga instructors and other participants in virtual yoga sessions

Exclusion criteria:

- Any severe psychiatric or medical comorbidity in the PI's judgment that would make study participation unsafe or not feasible.

Recruitment, Eligibility Screening, Enrollment, and Randomization

The office of CC Employee Health Plan will generate a list of potential participants ages 18-64 with a billing or ordering diagnosis of LBP (ICD-10 M54.5) in the last three years. CC EHP will then directly mail each potential participant identified a recruitment letter (*attached*) inviting the potential participant to contact the study team to learn more about the study. When an interested participant responds affirmatively in response to the recruitment letter by phone or e-mail, the study team member will communicate with the potential participant to perform a pre-screening interview (*attached*) for eligibility and assessment of the person's interest in participation. Interested participants will then be sent an Information Sheet (*attached*) in lieu of written consent via the participant's preferred method (e.g., e-mail, USPS or MyChart). The study team member will arrange a follow up call or IT approved, HIPAA-secure, virtual meeting with the person to re-confirm the participant's interest, confirm eligibility and review the study Information Sheet line by line allowing adequate time for questions about the study and assuring participant understanding through the "teach back" method. If the person verbally agrees to participate, the participant will complete a baseline survey via the participant's

preferred method (e.g., e-mail, USPS or MyChart) and subsequently randomize the participant to virtual yoga group or wait-list group. Participants will be informed that web links to virtual classes and to pre-recorded yoga classes will be sent to their specified e-mail address. The participant will be asked permission for instructors and other participants to view them during their virtual class. They will be asked to maintain the privacy and confidentiality of any comments made by others during class. They will also be asked their preferred method for follow-up communication going forward (e.g., e-mail, USPS, MyChart). The participant's preferred method will be used for follow-up questionnaires, and completion of yoga home practice logs. All study questionnaires will be stored in REDCap.

Information about the study will also be included in general newsletters, websites, other communications, and social media platforms that EHP and Cleveland Clinic use to target their employees and family members. We will use the Recruitment Flyer as advertisement in the following platforms:

- Internal websites of Cleveland Clinic: Intranet, DocCom, Connect Today
- EHP Website (https://employeehealthplan.clevelandclinic.org/)
- EHP newsletters, letters, and emails
- CC Corporate communications emails that are targeted to caregivers only (e.g., Caregiver Communications, Employee Wellness)
- CC employee designated areas, e.g., nurses' lounges, as approved by administration for identified area
- Social Media: CC Employee Facebook Page (closed group)

Members of the study team will also provide information about the study at routine clinical and employee meetings across the enterprise. If an interested person responds to either of these alternative methods, the same recruitment process will be followed. Study team members will routinely send EHP a list of enrolled participants to avoid redundant EHP recruitment mailings.

Screening and enrollment processes will all occur remotely and involve the following: (1) A telephone or IT approved, HIPAA-secure, virtual meeting platform where participants provide verbal consent for completion of eligibility screening through a standardized questionnaire; (2) if eligible, provision of information about the study with use of an IRB approved Information Sheet in lieu of informed consent with signature, answering all questions about the study, and assuring participant understanding through the "teach back" method.

Randomization occurs after administering the baseline survey (see Table 1 Data Collection Schedule). We will use a computerized randomization procedure built into our study management system, REDCap, to randomize each enrolled participant using a 1:1 ratio to yoga or wait-list control. Permuted variably-sized block randomization with block sizes of 6, 12 and 18 will be used.

Intervention and Comparator:

The study interventions start within approximately one week of baseline data collection and randomization. All participants throughout the entire 24-week study can continue to receive routine medical care including doctor visits and pain medication as prescribed.

The **hatha yoga intervention** is structured and reproducible. The full instructor manual and participant manual are available as hardcopy and online.[24] Originally, it was developed by an expert panel led by Rob Saper, MD MPH in 2007 and used in a pilot study of 30 participants with cLBP.[16] It was further refined in 2011 in a dosing study of 95 participants[17] and a larger non-inferiority trial of 320.[18] It is designed specifically for the yoga-naïve individual for effectiveness and safety in cLBP. Each class is 60 minutes long and will be delivered virtually to a maximum class size of 20. Yoga instructors will complete an 8-hour training on the protocol directed by our CC lead yoga therapist Judi Barr, E-RYT 500, C-IAYT. It begins with a relaxation exercise, yoga breathing exercises, and a brief discussion of yoga philosophy. The class proceeds with warm-up yoga exercises and then yoga postures. Yoga breathing is emphasized throughout each class. The class ends with a relaxation exercise. The 12 weeks are divided into four 3-week segments. Each segment is given a theme (e.g., "*Listening to the Wisdom of the Body*"). Participants are frequently advised to go slowly and carefully. The degree of difficulty of postures learned increases with each segment. For each segment, the participants gradually learn a sequence of 12–15 poses. The protocol provides variations and uses various aids (e.g., chair, strap) to accommodate a range of physical abilities. Participants are strongly encouraged to practice at home for 30 minutes daily on days which they do not attend yoga class. To facilitate home practice, participants will be given a free yoga mat, participant manual, and provided access to Webex, virtual classes led by the study instructors. Classes will be overseen by Cleveland Clinic's IT Video Conferencing team to manage the focus in 'Webex Layout' to view only instructor activity (PIN instructors to their activity stage) during class and mute participants during class time. Webex pre-recorded class links for participant practice between live classes will be stored on a secure Cleveland Clinic server for 'Videos on Demand,' which is an externally accessible, unlisted link that will be password protected. A study member will provide the password for the class link to specific class participants to view for weekly practice using participant registration captured in REDCap. Ten percent of online yoga classes will be reviewed for fidelity to the protocol using a checklist. A 12-week follow-up phase consisting of larger optional classes for maintenance will follow.

Individuals randomized to the wait list control will be offered non-study yoga classes after 24-weeks. They will also receive a free yoga mat and access to pre-recorded online videos.

Table 1: Data Collection Schedule

Data Collection	Eligibility Screening	Baseline	Week 6	Week 12	Week 24
Eligibility Screening	X				
Low Back Pain History		X			
Sociodemographics		X			
Pain Intensity		X	X	X	X
Roland-Morris Disability Scale		X	X	X	X
Pain Interference		X	X	X	X
Pain Medications		X	X	X	X
Satisfaction with Care		X	X	X	X
Global Improvement			X	X	X
Adverse Effects			X	X	X
Home Practice*			X	X	X

* Weekly diary for participants taking yoga classes

Outcome Measures

The co-primary outcome measures are (1) average pain intensity in previous week on an 11-point numerical rating scale where 0 is no pain and 10 is the worst pain possible, and (2) back-related function using the 23-point modified Roland Morris Disability Questionnaire (RMDQ). The main time point of interest is 12 weeks. Secondary outcomes include analgesic pain medication. This will be collected using several methods: (1) Self-report of all prescription and over the counter pain medications taken in the previous week including dosages and frequencies. (2) Mean morphine milligram equivalents (MME) taken per day per patient over the previous three months based on filled prescriptions, available through the EHP prescription database. Additional secondary outcomes include overall improvement by self-report (6-point Likert scale, 0 = very worse to 6 = very improved) and pain interference.

Implementation outcomes derived from study records will include rate of recruitment, proportion enrolled of total population contacted, attendance to yoga classes, home practice, study retention, missing data, and intervention costs. Acceptability will be measured by participant satisfaction with treatment (5-point Likert scale, 0 = very dissatisfied to 5 = very satisfied) and answers to open-ended questions about the effect of yoga, facilitators, and barriers. EHP will assist gathering utilization and medical expenditure data (admissions, visits, imaging, procedures, and total and back-specific costs of care). Adverse events will be collected from questionnaires and direct reports to study staff.

Data Analysis

The primary analysis will follow the intention-to-treat principle. Baseline characteristics will be summarized with appropriate descriptive statistics and will be compared between the two groups using t-test, Wilcoxon rank sum test, or chi-squared test as appropriate. Linear mixed-effects models will be used to analyze the co-primary outcomes, changes in pain numerical rating scale and the modified Roland Morris Disability Questionnaire. Each model will include time, group, their interaction, and baseline score. Any potential subject characteristics with significant differences at baseline will also be included in the model as potential confounders. A random intercept at the subject level will be included to account for the clustered data structure. Based on the model, the primary comparison will be the change from baseline between the two groups at week 12. Comparisons at other time points will be considered as secondary.

We will also perform multiple sensitivity analyses. A per-protocol analysis will be performed by replacing the binary group indicator with the number of yoga sessions attended (0 for the control participants and the exact number of classes attended by the yoga participants). Missing data are assumed missing at random in the primary analyses. We will also perform multiple imputation and analyze the imputed data.

Secondary outcomes will be analyzed similarly as above. Dichotomous or ordinal outcomes will be analyzed using generalized linear mixed-effects models. Adverse events will be summarized as frequencies and percentages and compared using the chi-squared test. All analyses will be conducted in R-studio (Boston, MA).

Sample size and power calculations. We assumed a minimal clinically significant decrease in pain intensity and Roland Morris based on the literature as 2.0 and 3.0 points, respectively. Standard deviations for these two outcomes based on previous studies are approximately 2.0 and 5.0, respectively. Given the two co-primary outcomes we will use an alpha=0.025. A conservative 20% attrition rate was assumed. We assumed no change in the control group. Given these parameters, a total sample size of 140 participants is estimated to provide 99% and 82% statistically and clinically significant differences in pain intensity and Roland Morris, respectively.

Implementation outcomes such as recruitment rates, yoga class attendance, home practice, study retention, missing data, implementation costs, and participant satisfaction will be summarized with descriptive statistics. Change in satisfaction between the Yoga Now and Yoga Later groups will be analyzed as described above for secondary outcomes. Responses to open-ended questions about the effect of yoga, facilitators and barriers will be analyzed using rapid thematic analysis.[25]

The Principal Investigator will identify and invite up to two (2) employees from the Clinic Diversity Office to complete scripted interviews conducted by study team members. The purpose of this interview is to understand if our study was representative of the Cleveland

Clinic employee health plan population and to better understand barriers when working within an employee population.

Up to four (4) interviews will be conducted by study personnel with yoga study instructors as related to their experience as instructors. The purpose of this is to understand what challenges they had while conducting the study and how to facilitate those challenges in future remote studies.

All interviews will be conducted and recorded via the secure Cleveland Clinic Team's app at which time the interviewees will be verbally consented to agree to being recorded. Recordings will be deleted after analysis is completed.

Cost-benefit from the perspective of the payer will be conducted by comparing incremental costs of the Yoga Now group compared to the Yoga Later group over the 24-week study period. For Yoga Now, the incremental costs will only include direct medical costs (direct intervention costs plus ongoing direct medical utilization costs). For the Yoga Later, it will be only direct medical utilization costs. Combined LBP and non-LBP-related healthcare utilization will be used for all of these analyses. Sensitivity analyses using LBP-related utilization only will also be performed. All cost effectiveness analyses will use the ITT principle.

We will compare aggregate demographic and socio demographics information on the entire employee health population (EHP) for comparisons with aggregate data of the study population to see if they are representative of the EHP population in age, gender, race, level of education, and employment status. We will use chi-square analysis to determine if there are significant differences between the employee population and our study sample. We will not document this information in REDCap and we will not report individual data.

Table 2. EHP Data Collection

Demographics & Sociodemographics	For comparison with Study Population
Age	All Age Ranges
Gender	Male / Female
Race	American Indian or Alaska Native, Asian, Black or African American, Native Hawaiian or Other Pacific Islander, White, Other
Level of Education	High School, GED certificate, Technical School, College, Masters, Doctorate
Employment Status	Active vs Non-Active

Confidentiality

All study data extracted from EPIC will be de-identified, stored and secured in the Cleveland Clinic server network. Only the study team will have access to the data, and if shared, using a password protected Excel spreadsheet PHI that could identify a participant will be removed or changed before files are shared with other researchers or results are made public. Data analysts' will not be given access to PHI or other identifiers. Outcomes data will be reported in aggregate, not by or with personal identifiers. We will follow Cleveland Clinic's strict policies to maintain privacy and confidentiality. The data will be maintained for a period of 6 years after completion of the study.

References

1. Hartvigsen J, Hancock MJ, Kongsted A, et al. What low back pain is and why we need to pay attention. *Lancet.* 2018;391(10137):2356-2367.

2. Licciardone JC. The epidemiology and medical management of low back pain during ambulatory medical care visits in the United States. *Osteopath Med Prim Care.* 2008;2:11.

3. Dieleman JL, Cao J, Chapin A, et al. US Health Care Spending by Payer and Health Condition, 1996-2016. *JAMA.* 2020;323(9):863-884.

4. Carey K, Ameli O, Garrity B, et al. Health insurance design and conservative therapy for low back pain. *Am J Manag Care.* 2019;25(6):e182-e187.

5. Qaseem A, Wilt TJ, McLean RM, Forciea MA, Clinical Guidelines Committee of the American College of P. Noninvasive Treatments for Acute, Subacute, and Chronic Low Back Pain: A Clinical Practice Guideline from the American College of Physicians. *Annals of internal medicine.* 2017;166(7):514-530.

6. Chou R, Deyo R, Friedly J, et al. Nonpharmacologic Therapies for Low Back Pain: A Systematic Review for an American College of Physicians Clinical Practice Guideline. *Ann Intern Med.* 2017;166(7):493-505.

7. Garland EL, Brintz CE, Hanley AW, et al. Mind-Body Therapies for Opioid-Treated Pain: A Systematic Review and Meta-analysis. *JAMA Intern Med.* 2019.

8. Herman PM, McBain RK, Broten N, Coulter ID. Update of Markov Model on the Cost effectiveness of Nonpharmacologic Interventions for Chronic Low Back Pain Compared to Usual Care. *Spine (Phila Pa 1976).* 2020;45(19):1383-1385.

9. Chou R, Deyo R, Friedly J, et al. Systemic Pharmacologic Therapies for Low Back Pain:

A Systematic Review for an American College of Physicians Clinical Practice Guideline. *Ann Intern Med.* 2017;166(7):480-492.

10. Sherry TB, Sabety A, Maestas N. Documented Pain Diagnoses in Adults Prescribed Opioids: Results From the National Ambulatory Medical Care Survey, 2006-2015. *Ann Intern Med.* 2018;169(12):892-894.

11. Han B, Compton WM, Blanco C, Crane E, Lee J, Jones CM. Prescription Opioid Use, Misuse, and Use Disorders in U.S. Adults: 2015 National Survey on Drug Use and Health. *Ann Intern Med.* 2017;167(5):293-301.

12. Sherman KJ, Cherkin DC, Erro J, Miglioretti DL, Deyo RA. Comparing yoga, exercise, and a self-care book for chronic low back pain: a randomized, controlled trial. *Ann Intern Med.* 2005;143(12):849-856.

13. Sherman KJ, Cherkin DC, Wellman RD, et al. A randomized trial comparing yoga, stretching, and a self-care book for chronic low back pain. 2011:2019-2026.

14. Williams K, Abildso C, Steinberg L, et al. Evaluation of the effectiveness and efficacy of Iyengar yoga therapy on chronic low back pain. *Spine.* 2009;34(19):2066-2076.

15. Tilbrook HE, Cox H, Hewitt CE, et al. Yoga for chronic low back pain: a randomized trial. *Ann Intern Med.* 2011;155(9):569-578.

16. Saper RB, Sherman KJ, Cullum-Dugan D, Davis RB, Phillips RS, Culpepper L. Yoga for chronic low back pain in a predominantly minority population: a pilot randomized controlled trial. *Altern Ther Health Med.* 2009;15(6):18-27.

17. Saper RB, Boah AR, Keosaian J, Cerrada C, Weinberg J, Sherman KJ. Comparing once- versus twice-weekly yoga classes for chronic low back pain in predominantly low income minorities: a randomized dosing trial. *Evid Based Complement Alternat Med.* 2013:658030.

18. Saper RB, Lemaster C, Delitto A, et al. Yoga, Physical Therapy, or Education for Chronic Low Back Pain: A Randomized Noninferiority Trial. *Ann Intern Med.* 2017;167(2):85-94.

19. Wieland LS, Skoetz N, Pilkington K, Vempati R, D'Adamo CR, Berman BM. Yoga treatment for chronic non-specific low back pain. *Cochrane Database Syst Rev.* 2017;1:CD010671.

20. Proctor EK, Powell BJ, McMillen JC. Implementation strategies: recommendations for specifying and reporting. *Implement Sci.* 2013;8:139.

21. Deyo RA, Dworkin SF, Amtmann D, et al. Report of the NIH Task Force on Research Standards for Chronic Low Back Pain. *Spine J.* 2014.

22. Powell BJ, Waltz TJ, Chinman MJ, et al. A refined compilation of implementation strategies: results from the Expert Recommendations for Implementing Change (ERIC) project. *Implement Sci.* 2015;10:21.

23. Curran GM, Bauer M, Mittman B, Pyne JM, Stetler C. Effectiveness-implementation hybrid designs: combining elements of clinical effectiveness and implementation research to enhance public health impact. *Med Care.* 2012;50(3):217-226.

24. Saper RB, Sherman KJ, Delitto A, et al. Yoga vs. physical therapy vs. education for chronic low back pain in predominantly minority populations: study protocol for a randomized controlled trial. *Trials.* 2014;15:67.

25. Hsieh HF, Shannon SE. Three approaches to qualitative content analysis. *Qual Health Res.* 2005;15(9):1277-1288.

26. Wu, Aimin et al. Global low back pain prevalence and years lived with disability from 1990 to 2017: estimates from the Global Burden of Disease Study 2017. *Annals of translational medicine* vol. 8,6 (2020): 299. doi:10.21037/atm.2020.02.175

27. Finley CR, Chan DS, Garrison S, Korownyk C, Kolber MR, Campbell S, Eurich DT, Lindblad AJ, Vandermeer B, Allan GM. What are the most common conditions in primary care? Systematic review. Can Fam Physician. 2018 Nov;64(11):832-840. PMID: 30429181; PMCID: PMC6234945.

28. GBD 2015 Disease and Injury Incidence and Prevalence Collaborators. Global, regional, and national incidence, prevalence, and years lived with disability for 310 diseases and injuries, 1990-2015: a systematic analysis for the Global Burden of Disease Study 2015. Lancet. 2016 Oct 8;388(10053):1545-1602. doi: 10.1016/S0140-6736(16)31678-6. Erratum in: Lancet. 2017 Jan 7;389(10064):e1. PMID: 27733282; PMCID: PMC5055577.

29. Sherry, T. B., Sabety, A., & Maestas, N. (2018). Documented pain diagnoses in adults prescribed opioids: Results from the national ambulatory medical care survey, 2006-

2015. Annals of Internal Medicine, 169(12), 892–894. https://doi.org/10.7326/M18-0644

30. Centers for Disease Control and Prevention. 2018 Annual Surveillance Report of Drug-Related Risks and Outcomes — United States. Surveillance Special Report 2pdf icon. Centers for Disease Control and Prevention, U.S. Department of Health and Human Services. Published August 31, 2018.

31. Singh, Vanila M., et al. (2019). Pain Management Best Practices Inter-Agency Task Force Report: Updates, Gaps, Inconsistencies, and Recommendations. Final Report. Department of Health and Human Services, Washington, DC
