# Supplementary material for: Effectiveness of Virtual Yoga for Chronic Low Back Pain: A Randomized Clinical Trial
Source: JAMA Netw Open. 2024 Nov 1;7(11):e2442339. doi: 10.1001/jamanetworkopen.2024.42339 (PMC11530940; doi:10.1001/jamanetworkopen.2024.42339)
Supplement: Supplement 2. — eTable 1. Yoga Class Format eTable 2. Sensitivity Analysis of Primary Outcomes Using Multiple Imputation eTable 3. Participants Experiencing Clinically Important Changes in Co-Primary Outcomes (30% or Greater Reduction From Baseline) [file jamanetwopen-e2442339-s002.pdf]

Supplementary Online Content

Tankha H, Gaskins D, Shallcross A, et al. Effectiveness of virtual yoga for chronic low back pain: a randomized clinical trial. *JAMA Netw Open*. 2024;7(11):e2442339.
doi:10.1001/jamanetworkopen.2024.42339

eTable 1. Sensitivity Analysis of Primary Outcomes Using Multiple Imputation

eTable 2. Participants Experiencing Clinically Important Changes in Co-Primary Outcomes (30% or Greater Reduction From Baseline)

This supplementary material has been provided by the authors to give readers additional information about their work.

© 2024 Tankha H et al. *JAMA Network Open*.

eTable 1. Sensitivity Analysis of Primary Outcomes Using Multiple Imputation

		Mean change from baseline (95% CI)					
**Outcome Measure**	Mean baseline value (SE)	6 weeks (95% CI)	*p* value	12 weeks (95% CI)	*p* value	24 weeks (95% CI)	*p* value
**Pain Intensity‡**							
Yoga Now	5.7 (0.2)	-1.4 (-1.9 to -0.8)		-1.7 (-2.2 to -1.1)		-2.1 (-2.6 to -1.6)	
Yoga Later	5.8 (0.2)	-0.2 (-0.8 to 0.3)		-0.3 (-0.9 to 0.2)		-0.2 (-0.7 to 0.4)	
Between-group mean difference		-1.1 (-1.9 to -0.3)	.005	-1.3 (-2.1 to -0.6)	< .001	-2.0 (-2.7 to -1.2)	< .001
**Back-related Function (RMDQ)§**							
Yoga Now	11.7 (0.6)	-2.7 (-3.9 to -1.5)		-3.7 (-4.9 to -2.5)		-5.6 (-7.2 to -4.0)	
Yoga Later	12.4 (0.6)	-0.6 (-1.8 to 0.7)		-1.7 (-2.8 to -0.7)		-1.7 (-2.8 to -0.6)	
Between-group mean difference		-2.1 (-3.8 to -0.4)	.01	-1.9 (-3.6 to -0.3)	0.02	-3.9 (-5.8 to -2.0)	< .001

RMDQ = Roland Morris Disability Questionnaire
‡Measured using an 11-point numerical rating scale for average pain intensity in the previous week, with 0 indicating no pain and 10 indicating worst pain possible.
§ Scores range from 0 to 23, with higher scores representing poorer function.

© 2024 Tankha H et al. *JAMA Network Open*.

eTable 2. Participants Experiencing Clinically Important Changes in Co-Primary Outcomes (30% or Greater Reduction From Baseline)

	6 weeks		12 weeks		24 weeks	
	Yoga Now	Yoga Later	Yoga Now	Yoga Later	Yoga Now	Yoga Later
**Observed data**						
Pain Intensity‡	30.1%	14.5%	38.0%	20.3%	36.6%	13.0%
RMDQ§	33.8%	13.0%	42.3%	24.6%	47.9%	26.1%
**Imputed data**						
Pain Intensity‡	42.3%	22.3%	52.4%	23.5%	53.5%	16.2%
RMDQ§	44.2%	18.8%	55.2%	30.7%	71.0%	33.3%

RMDQ = Roland Morris Disability Questionnaire
‡ Measured using an 11-point numerical rating scale for average pain intensity in the previous week, with 0 indicating no pain and 10 indicating worst pain possible.
§ Measure of back-related function with scores ranging from 0 to 23, with higher scores representing poorer function.

© 2024 Tankha H et al. *JAMA Network Open*.
