# Supplementary material for: Effectiveness of Virtual Yoga for Chronic Low Back Pain: A Randomized Clinical Trial
Source: JAMA Netw Open. 2024 Nov 1;7(11):e2442339. doi: 10.1001/jamanetworkopen.2024.42339 (PMC11530940; doi:10.1001/jamanetworkopen.2024.42339)
Supplement: Supplement 4. — Data Sharing Statement [file jamanetwopen-e2442339-s004.pdf]

Data Sharing Statement

Tankha. Effectiveness of Virtual Yoga for Chronic Low Back Pain. *JAMA Netw Open*. Published November 01, 2024. doi:10.1001/jamanetworkopen.2024.42339

Data
Additional Information: ClinicalTrials.gov (NCT05319691)
Data available: Yes
Data types: Deidentified participant data, Data dictionary
How to access data: saperr@ccf.org
When available: With publication

Supporting Documents
Document types: None

Additional Information
Who can access the data: researchers whose proposed use of the data has been approved
Types of analyses: for any purpose
Mechanisms of data availability: with a signed data access agreement
